# Quantitative fluorescent profiling of VEGFRs reveals tumor cell and endothelial cell heterogeneity in breast cancer xenografts

**DOI:** 10.1002/cam4.188

**Published:** 2014-01-22

**Authors:** Princess I Imoukhuede, Aleksander S Popel

**Affiliations:** 1 Department of Bioengineering, University of Illinois at Urbana ChampaignUrbana, Illinois, 61801; 2 Department of Biomedical Engineering, School of Medicine, Johns Hopkins UniversityBaltimore, Maryland, 21205

**Keywords:** Biomarker, endothelial cells, heterogeneity, personalized medicine, proteomics, quantitative flow cytometry, receptor localization, vascular endothelial growth factor receptors, xenograft

## Abstract

Plasma membrane-localized vascular endothelial growth factor receptors (VEGFR) play a critical role in transducing VEGF signaling toward pro and antiangiogenic outcomes and quantitative characterization of these receptors is critical toward identifying biomarkers for antiangiogenic therapies, understanding mechanisms of action of antiangiogenic drugs, and advancing predictive computational models. While in vitro analysis of cell surface-VEGFRs has been performed, little is known about the levels of cell surface-VEGFR on tumor cells. Therefore, we inoculate nude mice with the human triple-negative breast cancer, MDA-MB-231, cell line; isolate human tumor cells and mouse tumor endothelial cells from xenografts; and quantitatively characterize the VEGFR localization on these cells. We observe 15,000 surface-VEGFR1/tumor endothelial cell versus 8200 surface-VEGFR1/tumor endothelial cell at 3 and 6 weeks of tumor growth, respectively; and we quantify 1200–1700 surface-VEGFR2/tumor endothelial cell. The tumor cell levels of VEGFR1 and VEGFR2 are relatively constant between 3 and 6 weeks: 2000–2200 surface-VEGFR1/tumor cell and ∼1000 surface-VEGFR2/tumor cell. Cell-by-cell analysis provides additional insight into tumor heterogeneity by identifying four cellular subpopulations based on size and levels of cell membrane-localized VEGFR. Furthermore, when these ex vivo data are compared to in vitro data, we observe little to no VEGFRs on MDA-MB-231 cells, and the MDA-MB-231 VEGFR surface levels are not regulated by a saturating dose of VEGF. Overall, the quantification of these dissimilarities for the first time in tumor provides insight into the balance of modulatory (VEGFR1) and proangiogenic (VEGFR2) receptors.

## Introduction

Vascular endothelial growth factor (VEGF) signaling involves the binding of VEGF ligand to its receptors: VEGFR1, VEGFR2, VEGFR3, neuropilin-1 (NRP1) and NRP2, resulting in angiogenesis, vasculogenesis, lymphangiogenesis, and other changes in vascular function including: maturation, branching, development, and increased permeability 1. As a key regulator of tumor angiogenesis 2,3, VEGF is enriched in cancer patient plasma and serum samples 4–7, and the approach of sequestering VEGF to attenuate signaling and prevent abnormal vascularization 8,9 in the form of antibodies, small molecule tyrosine kinase inhibitors, and peptides 10–12 has been applied toward treating metastatic breast cancer, metastatic colorectal cancer, and glioblastoma multiforme—the most common and most aggressive form of brain cancer 8,13. Despite significant progress into antiangiogenic therapy, anti-VEGF therapy has had only moderate effects on patient survival, ultimately leading to antiangiogenic resistance and nonresponsiveness 14. Furthermore, some animal models have shown that anti-VEGF therapy can increase tumor invasion and metastasis. These antiangiogenic clinical challenges have been attributed to inadequate dosing, duration, delivery, and a need to identify accurate biomarkers of the heterogeneity in the vascular microenvironment 15,16. Systems biology offers unique approaches to analyze the tumor system and surmount these challenges through the coupling of sensitive measurements of the vascular microenvironment with computational modeling. This bimodal approach, experimental and computational, provides platforms for describing the tumor microenvironment, testing therapeutic conditions on multiple scales, and predicting optimal treatment strategies, thus advancing preclinical understanding of tumor progression and metastasis.

Multiscale computational models, based on mass-action kinetics of the VEGF-VEGFR signaling axis, have predicted the distribution of VEGF in the body upon administration of the anti-VEGF recombinant humanized monoclonal antibody, bevacizumab 17–19 and the VEGF Trap in mice 20. We have further advanced these computational models by experimentally determining and incorporating quantitative measurements of average endothelial and tumor VEGFR levels. This modeling provided a physiological framework for identifying the optimal drug and tumor properties for an anti-VEGF agent 21. The incorporation of experimentally determined VEGFR surface levels into a computational model significantly affected the predicted systemic and tumor distributions of VEGF by up to sixfold 21. These models thus predict that systemic and tumor VEGF levels strongly depend on VEGFR levels and a possible strategy for decreasing VEGF levels may lie in VEGFR modulation. Therefore, continued advancement of antiangiogenic therapies for cancer requires profiling of angiogenic receptors on modeled tissues.

Plasma membrane proteomic profiling offers a useful approach to both quantify receptor levels and characterize tumor heterogeneity. We have recently optimized a state-of-the-art fluorescence approach toward quantitatively profiling plasma membrane-localized, endothelial VEGFRs: in vitro 22 and on both skeletal muscle, ex vivo 23 and ischemic skeletal muscle, ex vivo 24. Additionally, we have developed tools for multiplexed receptor profiling 25. Our in vitro analysis revealed significant VEGF-mediated shifts in endothelial heterogeneity 22. Our ex vivo, endothelial skeletal muscle studies identified endothelial heterogeneity in two different mouse strains, whose extensively studied differences in vascular properties and ischemic response may serve as proxies for human population variability 26–31. Our hindlimb ischemia studies revealed significant downregulation of VEGFR2, 3 days after femoral artery ligation and an upregulation in VEGFR1, 10 days after ischemia induction; these changes are observed on the ischemic endothelial cells 24. These changing receptor dynamics on endothelium underscores a changing angiogenic environment requiring similarly dynamic therapeutic strategies. Therefore, the development of improved receptor-targeted therapies requires continued efforts to dynamically map receptor surface distributions in vascular pathologies.

To this end, here, we couple sensitive cell isolation of tumor cells and tumor endothelial cells (tEC) with quantitative, plasma membrane proteomic profiling to map and interpret heterogeneity defined by VEGFR membrane localization. Additionally, we contextualize these results through comparison to tumor and endothelial cell models, in vitro. These studies for the first time, give quantitative insight into the balance of cell surface angiogenic receptors at early and late stages of tumor xenograft development. Thus, they provide the quantitative data needed to identify biomarkers of antiangiogenic therapies 32 and advance systems biology approaches of studying and interpreting tumor development.

## Materials and Methods

### Cell culture

Human umbilical vein endothelial cells (HUVEC) are acquired from individual donors (Lonza, Walkersville, MD and Stem Cell Technologies, Vancouver, Canada). The endothelial cells are maintained in endothelial cell growth medium-2 (EGM-2), supplemented by the EGM-2 SingleQuot Kit (Lonza). Breast cancer cells MDA-MB-231 are kindly provided by Dr. Z. M. Bhujwalla (Johns Hopkins University) with the following details about the cell line: MDA-MB-231 breast cancer cells are purchased from the American Type Culture Collection (ATCC) and used within 6 months of obtaining them from ATCC; the cell line is tested and authenticated by ATCC by two independent methods; the ATCC cytochrome C oxidase I polymerase chain reaction (PCR) assay and short tandem repeat profiling using multiplex PCR. MDA-MB-231 cells are maintained in high glucose Dulbecco's modified Eagle medium (DMEM) containing 10% fetal bovine serum (Invitrogen, Carlsbad, CA) and 1% Penicillin–Streptomycin (Invitrogen). Cells are grown at 37°C in 95% air, 5% CO2. Cells are grown to confluence before use and HUVECs are only used through passage 6.

### Cell dissociation and in vitro receptor quantification

For routine cell culture, cells are detached from flasks using 0.25% trypsin (Invitrogen). We have previously shown that trypsin can affect the quantification of certain receptors 22; therefore, for in vitro quantitative flow cytometry, the nonenzymatic, cell dissociation solution (Millipore, Billerica, MA) is applied for 5–7 min at 37°C. Cells are resuspended in 10 mL FBS stain buffer (BD Biosciences, San Jose, CA), centrifuged at 300*g* for 4 min, supernatant is aspirated, and cells are resuspended in 10 mL FBS stain buffer. Cells are centrifuged and resuspended to a final concentration of 4 × 10^6^ cells/mL in FBS stain buffer. Quantitative flow cytometry on MDA-MB-231 cells and HUVECs, in vitro is performed as previously described 22.

### Growth factor application

Recombinant hVEGF-A_165_ (Shenandoah Biotechnology, Warmack, PA) is reconstituted with Dulbecco's phosphate-buffered saline (PBS) without calcium or magnesium (Invitrogen) at a concentration of 50 μg/mL and stored at −20°C. VEGF-A_165_ is applied for 5, 10, 15, and 30 min to determine the short-term effect on receptor density and 1 nmol/L VEGF-A_165_ is applied for 20–24 h, to determine the long-term effect of VEGF_165_ on receptor density. 1 nmol/L represents a saturating dose, given the VEGFR1 *K*_d_ of 16–30 pmol/L 33,34 and VEGFR2 *K*_d_ of 75–760 pmol/L 33,35, and our prior dose-response study showing upregulation of VEGFR1 and downregulation of VEGFR2 at this dose in HUVECs, human microvascular dermal endothelial cells, and human microvascular dermal lymphatic endothelial cells 22.

### Tumor xenografts

Animal protocols are approved by the Institutional Care and Use Committee at the Johns Hopkins Medical Institutions (JHMI). MDA-MB-231 breast cells are dissociated from flasks with TrypLE (Invitrogen), washed twice in PBS, and resuspended in DMEM. Mice are anesthetized using 0.125 mg acepromazine and 12.5 mg ketamine. Subsequently, 2 million cells/100-μL solution are injected into each side of the mammary fat pad of 7-week-old female, athymic NCr-*nu/nu* mice. Although athymic NCr-nu/nu mice are immunocompromised, lacking thymus gland, and thus do not express T cells, they express tumor-associated macrophages (TAMs) 36,37. The presence of TAMs is necessary for accurate profiling of angiogenesis within the tumor microenvironment as TAMs regulate tumor growth, invasion, metastasis, and angiogenesis 38–40. Tumors size is calculated by measuring the long (*l*) and short (*s*) axis of the ellipsoid tumor with a caliper and applying the following equation: *V* = *s *× *l*^2^.

### Endothelial cell isolation

Mice are euthanized with CO_2_ for ∼5 min, tumors are excised and placed in a 50 mL conical tube on ice containing cold Hanks balanced salt solution without magnesium and without chloride (Mediatech, Manassas, VA). Tumors are digested using procedures previously established for endothelial cell isolation from skeletal muscle 24,41. Briefly, each tumor is separately minced into 1-mm sections and added to freshly prepared 0.2% collagenase type IV filtered (Worthington Biochemical Corporation, Lakewood, NJ), which had been reconstituted in Hanks Balanced Salt Solution without calcium andmagnesium. Each individual tumor is digested for 30 min at 37°C with intermittent vortexting then passed through a 70 μm strainer (BD Bioscience, San Diego, CA). Cells from an individual tumor are centrifuged at 300*g* for 5 min and resuspended in 30 mL of 0.2 μm filtered isolation buffer, containing PBS without calcium and magnesium (Invitrogen), 2 mmol/L EDTA (Mediatech), and 0.1% BSA (Sigma-Aldrich, St. Louis, MO). Mouse tEC are isolated from the cell suspension using DSB-X (Invitrogen) biotinylated mouse CD31 antibody (eBioscience and BD Bioscience, San Diego, CA) and FlowComp Dynabeads (Invitrogen) according to the manufacturers' instructions. In this study, we only quantify VEGFR1 and VEGFR2, because the levels of these receptors are unchanged by the collagenase IV tissue dissociation; however, NRP1 is not quantified, because its surface levels are significantly decreased following collagenase IV treatment, possibly due to the presence of trypsin, which we have previously found to decrease NRP1 surface levels 22. Cell staining and flow cytometry are performed as we have previously described 22.

### Cell staining and flow cytometry

A volume of 25 μL aliquots of isolated cells ∼1 × 10^4^–1 × 10^5^ cells per tumor are added to tubes and are dually labeled with antibodies to CD34 and VEGFRs. As CD31 is also expressed on T cells, B cells, NK cells, macrophages/monocytes, granulocytes, and platelets, we label with 10 μL of mouse anti-CD34-FITC (BD Pharmingen, San Jose, CA). CD34 is expressed on endothelial cells, stem cells/precursors, mast cells, and neurons, the latter of which should be excluded by the prior CD31 magnetic bead separation 42,43. We also label with 10 μL mouse phycoerythrin (PE)-conjugated monoclonal antibody for the mouse endothelial cell isolate and 10 μL human PE-conjugated monoclonal antibody for the remaining cellular isolate at final concentrations of 14 μg/mL for VEGFR1 and VEGFR2 (R&D). Using human VEGFR antibodies excludes stromal cells from the quantification. The concentrations are reported to be saturating by the manufacturer, and we previously used anti-hVEGFR1-PE, anti-hVEGFR2-PE, anti-hVEGFR3-PE, and anti-hNRP1-PE (R&D Systems, Minneapolis, MN) at concentrations recommended by the manufacturer and independently confirmed those concentrations to be saturating 22. Tubes are protected from light and incubated for 40 min on ice. Cells are washed, centrifuged twice with 4 mL FBS stain buffer, and resuspended in 400 μL stain buffer.

As previously described, flow cytometry is performed on a FACSCalibur (BD Biosciences); CellQuest (BD Biosciences) software is used for data acquisition and FlowJo (Tree Star, Ashland, OR) is used for data analysis 22. Tubes are vortexted prior to placement in the flow cytometer. A total of 5000–10,000 cells are collected. Nonlabeled cells are analyzed to establish cellular autofluoresence conditions. PE-labeled cells are analyzed to establish PE fluorescence. Single cells are selected (gated) for CD34-FITC-positive cells (FL1 channel/FITC Fluorescence). Cells are further gated in the forward scatter and side scatter channels to select single-cell population.

### Optimizing quantification ex vivo

The precision and accuracy of quantitative flow cytometry has been rigorously tested 44–46. We have previously reported the optimization of this technique, confirming antibody specificity by quantifying labeling to porcine aortic endothelial cells stably expressing VEGFR2 and NRP1 22. We have previously established antibody saturation, monomeric antibody binding, and cell dissociation-receptor effects. We have also optimized this approach for ex vivo VEGFR quantification, determining that VEGFR1 and VEGFR2 surface levels are unaffected by our tissue digestion method 23. Furthermore, we have previously confirmed that our anti-CD31-magnetic bead isolation approach enriches endothelial cells through real-time quantitative RT-PCR comparison of total RNA isolation from both whole-cell preparation and CD31^+^ cell fraction 24.

### Statistical analysis: ensemble-averaged data

**Figure 1:**
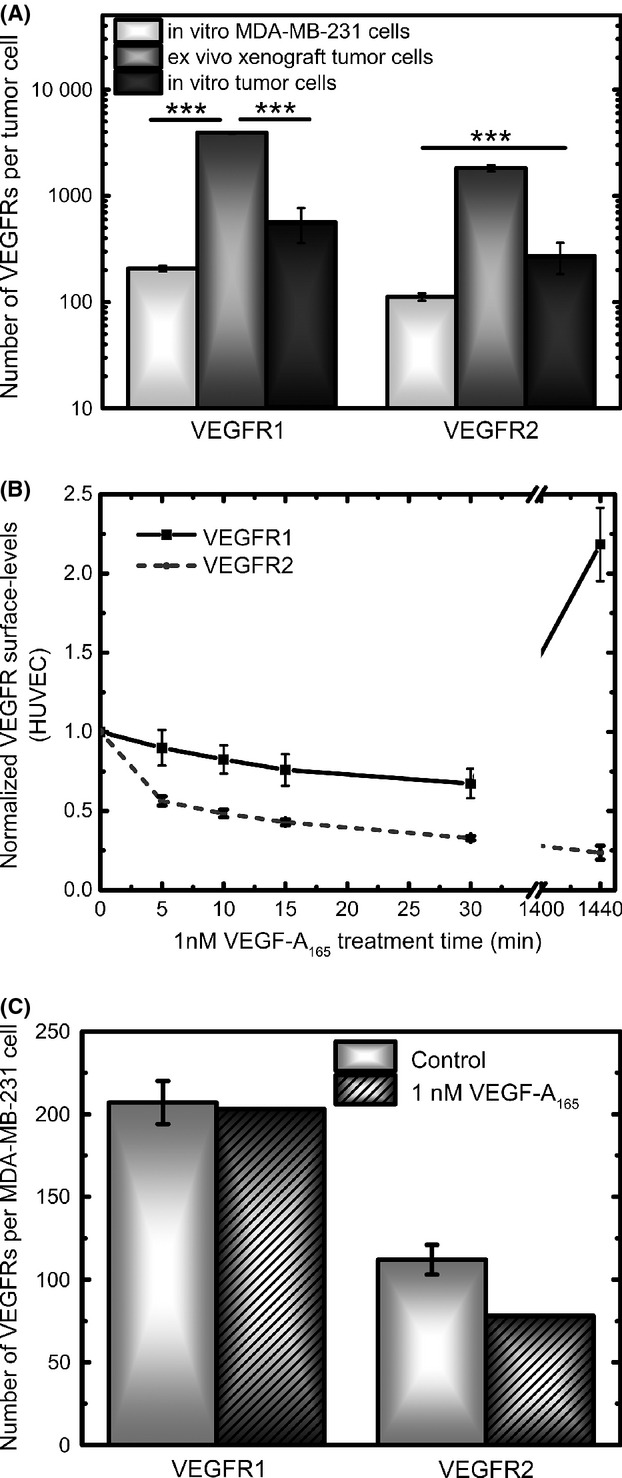
MDA-MB-231 and HUVEC cell characteristics, in vitro. (A) MDA-MB-231 cells show low levels of VEGFR1 and VEGFR2 in culture. Xenografts display significant upregulation of these receptors and when these tumor cells are returned to culture for 1 week, a downregulation of VEGFRs is seen. (B) HUVECs are responsive to VEGF-A_165_ in both the short-term and the long-term. Both VEGFR1 and VEGFR2 are downregulated within 30 min of 1 nmol/L VEGF-A_165_ treatment. After 24 h of 1 nmol/L VEGF-A_165_ treatment, VEGFR1 is significantly upregulated and VEGFR2 is further downregulated. (C) 1 nmol/L VEGF-A_165_ treatment does not significantly change the surface levels of VEGFRs on MDA-MB-231 cells. HUVEC, human umbilical vein endothelial cells; VEGFR, vascular endothelial growth factor receptor. *P < 0.05; **0.001< *P*<0.01; ***P < 0.001.

A PE calibration curve is generated from a sample of Quantibrite PE beads (BD Biosciences) on each experimental day. The Quantibrite PE beads are gated in the forward scatter and side scatter channels to select the bead population, as previously described 22. The geometric means 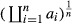 of the fluorescence intensities for the four bead peaks are obtained from the Quantibrite bead sample using the same compensation and voltage settings for acquiring cell fluorescence data, as previously described 22. These four fluorescence peaks corresponded to low (474 PE molecules/bead), medium-low (5359 PE molecules/bead), medium-high (23,843 PE molecules/bead), and high (62,336 PE molecules/bead) PE bead levels. The geometric mean of the fluorescence intensity for each bead is plotted versus the number of PE molecules per bead, and the calibration curve is fitted by linear regression *y* = *mx* + *b,* where “*x*” represents log_10_(PE molecules/bead), “*y*” represents log_10_(geometric mean fluorescence intensity/bead), “*m*” represents the slope of the PE-bead calibration curve, and “*b*” represents the *y*-intercept of the PE-bead calibration curve (Fig. 1C). This calibration curve is used to determine the number of PE molecules per cell, which corresponds to the number of receptors per cell. In order to obtain the numbers of receptors per cell, at least 10,000 cells are gated in the forward scatter and side scatter channels to select the single-cell population, “singlets,” and the geometric mean of the sample's PE fluorescence intensity is obtained, this value is converted to number of PE molecules/cell, which is approximately equal to the number of receptors/cell 45,47. Finally, the geometric mean of the sample's PE fluorescence intensity for nonlabeled cells is similarly converted, corresponding to autofluorescence, and these numbers of receptors/cell are subtracted from the geometric mean numbers of receptors/cell for the sample as shown: *R* = *R*_*s*_ − *R*_*a*_, where *R* corresponds to corrected number of receptors/cell, *R*_*s*_ corresponds to the geometric mean numbers of receptors/cell from labeled cells, and *R*_*a*_ corresponds to the geometric mean numbers of receptors/cell from nonlabeled cells (autofluorescence). The arithmetic mean, which we report, is generated by compiling multiple samples, thus giving the mean number of receptors/cell (Tables3 and 4). Values are expressed as mean ± standard error of the mean. Unless otherwise noted, *P* < 0.05 is considered statistically significant using the Student–Newman–Keuls analysis of variance.

### Statistical analysis: cell-by-cell data

Cell-by-cell data are analyzed by gating singlets in the forward scatter and side scatter channels, and PE fluorescence intensity for each gated cell is exported using FlowJo (Tree Star, Ashland, OR). Endothelial cell PE fluorescence intensity is converted into numbers of receptors per cell using the PE bead calibration obtained during that imaging session. Receptor densities are pooled and data greater than three standard deviations above the mean are excluded. The excluded data represent between 0.30 and 1.15% of the total data. Twelve different datasets are analyzed: tumor cells, tEC, and autofluorescence at weeks 3 and 6, and an appropriate distribution is fit to each. VEGFR1 and VEGFR2 are assumed to follow a multiple component mixture model, given the multiple-cell subpopulations observed by the data, while only one population is observed in the nonlabeled cell populations, or autofluorescence datasets. The log of the data follows a mixture of normal distributions, implying that the original data follow a lognormal distribution. The lognormal distribution is given as: 
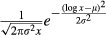
 (Eq. 1). The density of a random variable which is generated via a mixture model follows the form: 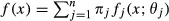. Here, *π*_*j*_ represents the probability of *x* coming from the *j*^th^ component (so 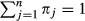), the *j*^th^ component has density *f*_*j*_ (*x*; *θ*_*j*_) and *θ*_*j*_ is the parameter vector for the corresponding density, and *n* represents the number of components 48. The mean, standard deviation, and density for each cell sub-population are determined using The R Project for Statistical Computing R and the “Mixtools” package 49,50. Although there are no known tests for goodness of fit for mixture models, we combined a qualitative assessment and statistical comparison to arrive at the optimal mixture models. Our qualitative assessment is performed by fitting two-components versus three-components. Figure S1 qualitatively displays that a two-component model does not represent the log-transformed data from tECs expressing VEGFR1 at week 3, while a three-component model captures each of the illustrated cell populations. The statistical goodness of fit is determined by applying the Kolmogorov–Smirnov (K-S) test: comparing the empirical distribution of the data with a theoretical cumulative distribution function. The null hypothesis of the test indicates that the empirical and the theoretical distribution are the same 51. The K-S test has an asymptotic power of 1, meaning any differences at all in the empirical and theoretical distributions will lead to a low *P*-value for large datasets, even if the practical differences between the two distributions are negligible. Given the size of our data, we expect low *P*-values, suggesting that the empirical and theoretical distributions are not equal. Therefore, we applied the K-S test to compare *P*-values for two-component versus three-component mixtures. Each of the two-component mixtures gave *P*-values less than 2.2 × 10^−16^; while the *P*-values for the three-component mixtures are significantly larger and are given in Table 1.

**Table 1:** Mixture model parameters for tumor cell and tumor endothelial cell distributions.

				Mean			Standard deviation			Density		
Receptor	Time	Sample	K-S test *P*	*μ*_1_	*μ*_2_	*μ*_3_	*σ*_1_	*σ*_2_	*σ*_3_	*π*_1_	*π*_2_	*π*_3_
VEGFR1	Week 3	tEC	0.0386	13,100	75,400	1100	2.27	1.45	2.18	0.83	0.11	0.06
		tumor	0.0507	2900	1300	10,000	1.57	1.46	2.69	0.73	0.15	0.12
	Week 6	tEC	0.0166	600	1300	10,600	1.46	2.32	2.66	0.52	0.36	0.12
		Tumor	6.50 × 10^−8^	3200	600	3000	1.55	1.68	4.62	0.71	0.18	0.11
VEGFR2	Week 3	tEC	0.0683	1300	1100	7500	2.23	1.52	2.75	0.44	0.43	0.13
		Tumor	0.0039	1400	2600	17,900	1.52	1.73	2.18	0.72	0.21	0.07
	Week 6	tEC	1.68 × 10^−4^	600	1500	16,200	1.48	2.59	1.99	0.61	0.34	0.05
		Tumor	1.86 × 10^−5^	1700	800	19,700	1.45	2.18	2.10	0.51	0.47	0.02

K-S, Kolmogorov–Smirnov; VEGFR, vascular endothelial growth factor receptor; tEC, tumor endothelial cell.

## Results

### VEGFRs are found on xenograft tumor cells

We have previously reported that MDA-MB-231 cells, a human, breast cancer cell line, have very little to no surface expression of VEGFR1 and VEGFR2, in vitro 22. However, VEGFRs are robustly expressed on human tumor cells from MDA-MB-231 tumor xenografts (Fig. 1A). Ensemble averaging of VEGFRs on xenografts indicates plasma membrane levels of 3900 VEGFR1/tumor cell and 1800 VEGFR2/tumor cell. When these extracted human tumor cells are returned to two-dimensional cell culture for 1 week, VEGFRs are significantly downregulated to 600 VEGFR1/tumor cell and 300 VEGFR2/tumor cell (Fig. 1A).

### VEGF does not upregulate VEGFRs, in vitro

Tumors are known to express high levels of VEGF 52, and we observe that 1 nmol/L VEGF-A_165_ can regulate in vitro endothelial expression of VEGFR in the short term (0–30 min), decreasing VEGFR1 surface levels by 25% and decreasing VEGFR2 surface levels by over 50% (Fig. 1B). However, we have previously shown 22 that long-term, 24 h, treatment of HUVECs with 1 nmol/L VEGF-A_165_ results in differential regulation of VEGFRs, upregulating VEGFR1 and downregulating VEGFR2 (Fig. 1B). We explored whether VEGFR surface localization would be similarly regulated by VEGF on MDA-MB-231 cells, in vitro. We treated MDA-MB-231 cells with the HUVEC-saturating dose of 1 nmol/L VEGF-A_165_ 22 for 24 h. The results show that 1 nmol/L VEGF-A_165_ is not sufficient to upregulate VEGFRs on MDA-MB-231 cells, in vitro (Fig. 1C).

As we observed significant expression of VEGFRs on tumor xenografts (Fig. 1A), we sought to examine how receptor surface expression compares in early-stage tumors and late-stage tumors. We inoculated MDA-MB-231 cells and extracted tumors at both 3 and 6 weeks. We observe that by 3 weeks of tumor growth, the tumor has reached a volume of 0.62 ± 0.17 cm^3^ and at 6 weeks the tumor volume is 1.5 ± 0.1 cm^3^. Our analysis of tumor xenografts includes the isolation of both the human tumor cells and the mouse tEC from the tumor.

### Establishing cell isolation via flow cytometry

**Figure 2:**
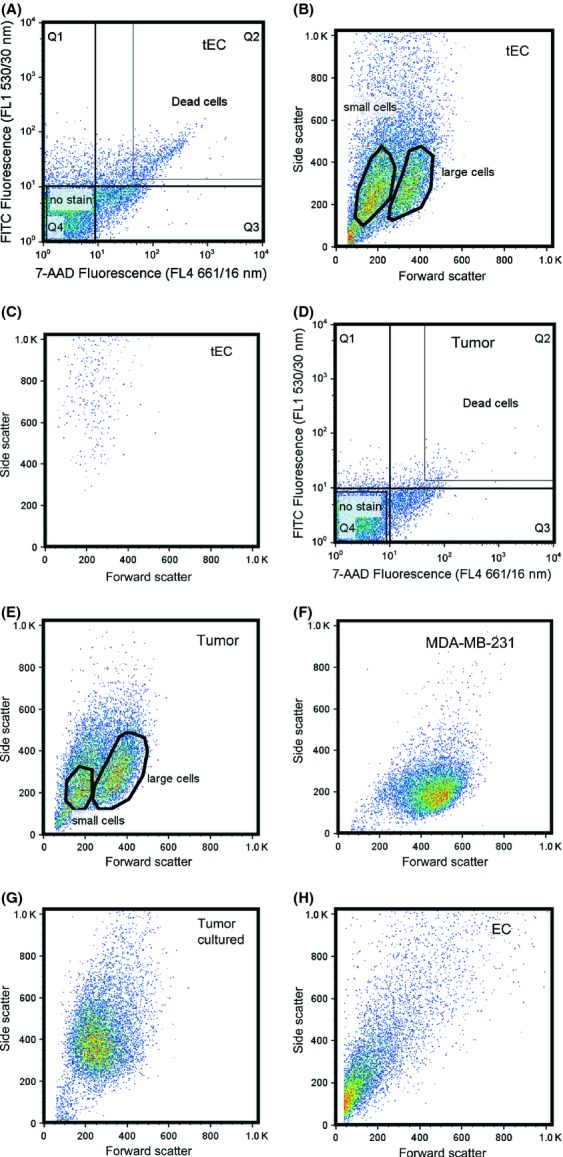
Flow cytometry dot plots. (A) A representative plot of cell viability, 7-aminoactinomycin D (7-AAD) staining, is shown of a tumor endothelial cell and (D) tumor cell population. Dead cells exhibit high-fluorescence intensity, and are shown in quadrant 2, Q2. Nonstained cells, or viable cells, are observed in Q4. (B and E) Selected, or “gated” nonstained (B) tumor endothelial cells (tEC) from “A” and (E) tumor cells from “D” are shown in the side scatter versus forward scatter plot. Two populations of cells are observed, both populations have similar granularity or side scatter, but differ in size or forward scatter. (C) The dead cells, observed in Q2 of (A) are gated and shown. They exhibit high granularity. (F) MDA-MB-231 cells, in vitro and (H) endothelial cells isolated from mouse skeletal muscle are represented by only one cell population. (G) When tumor cells extracted from xenografts are returned to culture for 1 week, only one cell population is observed.

Following xenograft dissociation, we stained cells with the viability dye 7-Aminoactinomycin D (7-AAD). Quadrant 4 (Q4) of the dot plot displays a selection, or “gating” of tEC that exclude the 7-AAD fluorophore, labeled as “no stain” within the dot plot, while nonviable cells are observed in the upper region of Q2 and are labeled as “dead cells” within the dot plot (Fig. 2A). These nonviable tEC display 10–100 times greater 7-AAD fluorescence compared to the viable tEC found in Q4 (Fig. 2A). When the live-cell population, Q4, is further analyzed for tumor endothelial cell sizing or forward scatter versus tumor endothelial cell granularity or side scatter (Fig. 2B), two tumor endothelial cell populations are apparent. When the nonviable tEC are analyzed for sizing and granularity (Fig. 2C), we observe that these tEC display significant granularity, consistent with prior flow cytometry observations of early apoptotic cells 53,54. Furthermore, we find that a majority of viable tEC do not overlap with nonviable tEC in the forward scatter versus side scatter plots, more precisely, >85% of tEC lie within a population distinct from nonviable cells (Fig. 2B). Therefore, we gate tEC based on their forward scatter and side scatter populations, not by 7-AAD exclusion. Applying the same live cell and dead cell gating to tumor cells reveals fewer dead tumor cells, <0.5% of the total observed tumor cell population (Fig. 2D), compared to ∼3.6% of the total observed tumor endothelial cell population (Fig. 2A). The “no stain” population in Q4 is also resolved as two populations (Fig. 2E). As we determined that the dead tumor cells lay mostly outside the live-tumor cell region, we gate the two populations of live tumor cells in our data analysis, rather than exclude dead tumor cells.

### Xenograft cells display heterogeneity in size and receptor density

**Figure 3:**
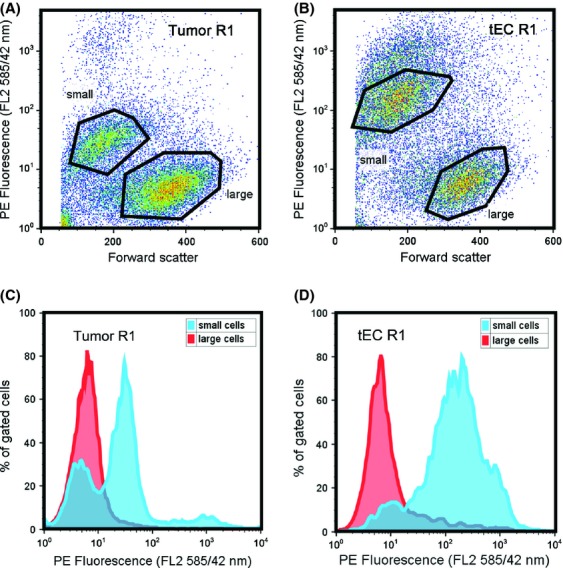
Flow cytometry characteristics of tumor and tumor endothelial cells. Representative flow cytometry dot plots of phycoerythrin (PE) fluorescence versus the sizing parameter, forward scatter reveal that (A) tumor cells and (B) tumor endothelial cells are characterized by two populations whose fluorescence does not scale with size. The large cells are represented by one, relatively low VEGFR-expressing population; and the small cells display multimodal distributions. This is illustrated in representative histogram plots from (C) tumor cells and (D) tumor endothelial cells that have been labeled with anti-VEGFR1-PE. VEGFR, vascular endothelial growth factor receptor.

The presence of two tumor cell and two tumor endothelial cell populations is consistent across xenografts from five different mice and is unique to the tumor xenografts. MDA-MB-231 cells do not exhibit this characteristic, in vitro (Fig. 2F), nor do the extracted xenograft tumor cells, following 1 week in culture (Fig. 2G). The dual population seen in the xenografts is also not inherent to primary tissue, as displayed in Figure 2H, where endothelial cells from skeletal muscle are shown to exist as a single population. When we further examine the fluorescence intensity of the two populations of tumor cells and tEC, we designate one population as “small cells,” due to their display of low forward scatter and the second population as “large cells,” due to their display of a higher forward scatter (Fig. 2E). To better examine these cells, we plot the fluorescence versus the relative sizing parameter (forward scatter). We find that fluorescence does not scale linearly with size (Fig. 3A and B). Dot plots representing PE fluorescence versus forward scatter for tumor cells (Fig. 3A) and tEC (Fig. 3B) labeled with VEGFR1-PE show that the small cells have higher average fluorescence compared to the large cells (Fig. 3A and B). These tendencies indicate that the two populations are unique and that the large cells do not simply represent doublet cells, as doublets would typically display twofold fluorescence intensity 55. When these small cells and large cells are further analyzed, as a histogram, we observe that both the small tumor cells (Fig. 3C) and small tEC (Fig. 3D) contain multiple-cell subpopulations. This is illustrated by the black arrowheads in Figure 3C and D. The large tumor cells (Fig. 3C) and large tEC (Fig. 3D) are represented by only one population of cells expressing low levels of VEGFRs, which is illustrated by the gray arrowheads in Figure 3C and D. These characteristics are shared across weeks 3 and 6 tumor cells and tEC labeled with VEGFR1 and VEGFR2.

### Characterizing the distributions: lognormal mixture model

**Figure 4:**
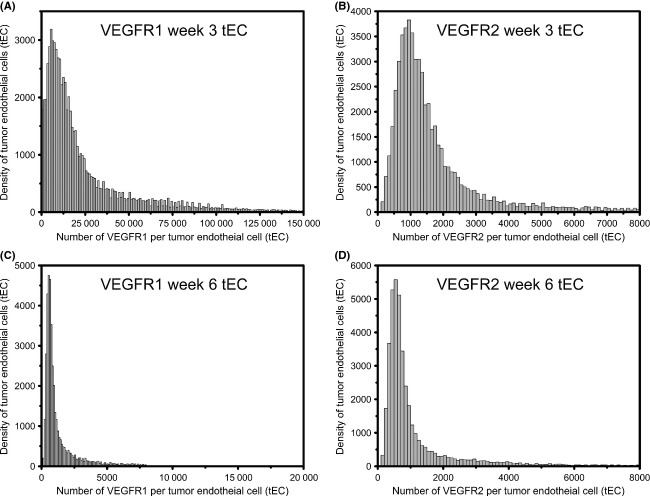
Mixture model distributions for tumor cells. Tumor cells at (A and B) week 3 and (C and D) week 6 of tumor growth. (A and C) Vascular endothelial growth factor receptor (VEGFR)1 and (B and D) VEGFR2 on the tumor cells are represented as a three-component mixture model of lognormal distributions.

**Figure 5:**
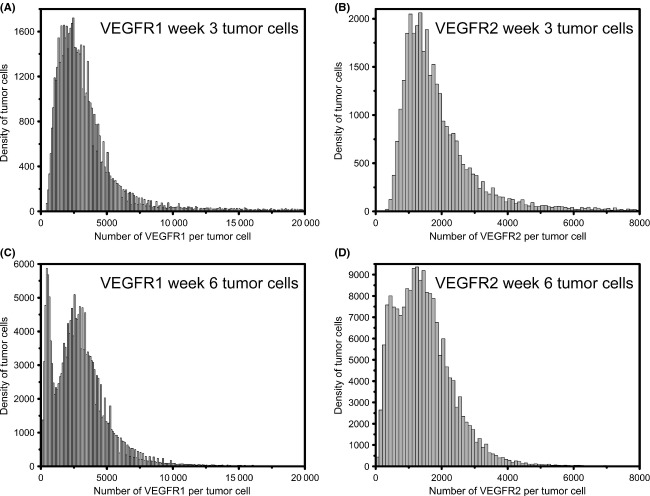
Mixture model distributions for tumor endothelial cells (tECs). tECs at (A and B) week 3 and (C and D) week 6 of tumor growth. (A and C) Vascular endothelial growth factor receptor (VEGFR)1 and (B and D) VEGFR2 on the tEC are represented as a three-component mixture model of lognormal distributions.

Given that multiple-cell subpopulations are seen across the small tumor cell and small tEC, we sought to better characterize these distributions using mixture modeling. The log of the data follows a mixture of normal distributions, implying that the original data follow a lognormal distribution. These are illustrated in Figures 4 and 5: where the VEGFR1 and VEGFR2 distributions represent three-component mixtures indicating that there are three-cell subpopulations.

### Tumor endothelial cell heterogeneity

Table 1 gives the estimates for the lognormal mixture model parameters. The columns have been ordered highest to lowest according to component weight: the cell population with the highest density is listed first (Table 1). For example, we see that at 3 weeks, early in tumor growth, of the three populations of tEC, 83% have mean VEGFR1 surface levels of ∼13,100 surface- VEGFR1/tEC; 11% present an average of ∼75,400 surface-VEGFR1/tEC; and 6% present ∼1100 surface-VEGFR1/tEC (Fig. 4A). In contrast, by week 6, the levels of VEGFR1 on tEC drop significantly, with 52% having only 600 surface-VEGFR1/tEC, 36% having only 1300 surface-VEGFR1/tEC, and 12% of cells presenting 10,600 VEGFR1/tEC (Fig. 4C). We see that at 3 weeks, a three-component mixture model for surface-VEGFR2 reveals low surface levels on tEC, with 44% presenting an average of 1300 surface-VEGFR2/tEC; 43% presenting 1100 surface-VEGFR2/tEC; and 13% presenting an average of 7500 surface-VEGFR2/tEC (Table 1).

### Tumor cell heterogeneity

**Figure 6:**
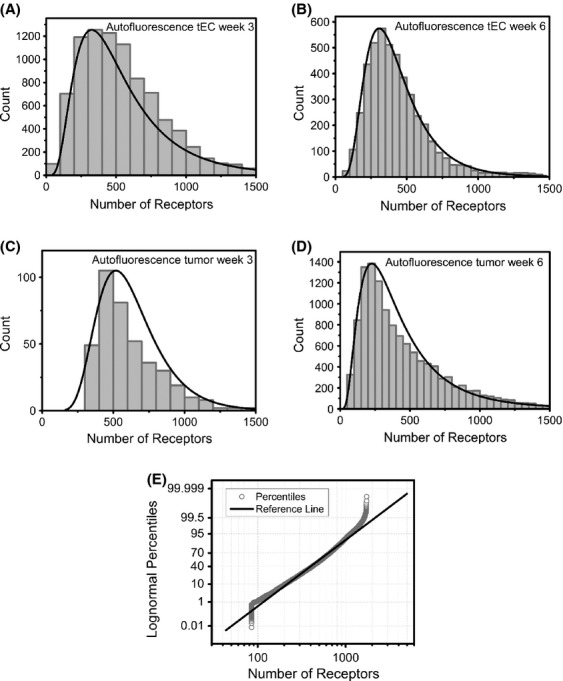
Nonlabeled cell/autofluorescence distributions. Lognormal distributions fit well to the (A) tumor endothelial cells at week 3 and (B) week 6, (C) tumor cells week 3 and (D) week 6. (E) A representative lognormal probability plot for tumor endothelial cells week 3 further shows that over 95% of the autofluorescence data are well fit by the lognormal distribution.

In general, we find that the levels of VEGFR1 on tumor cells are moderately low at both 3 weeks (Fig. 5A) and 6 weeks (Fig. 5C). Although, the distributions at both time points are qualitatively different, there is a quantitative similarity in averages: ∼70% of week 3 tumor cells have an average of 2900 surface-VEGFR1/tumor cell and ∼70% of week 6 tumor cells have an average of 3200 surface-VEGFR1/tumor cell (Table 1, Fig. 5A and C). The subpopulations representing the remaining cells, represent a small fraction of the total cell population, with <20% having surface levels of <1500 VEGFR1 at both time points and∼11% having surface levels of 10,000 VEGFR1 at week 3 (Fig. 5A) and 3000 VEGFR1 at week 6 (Fig. 5C). VEGFR2 surface levels on tumors, display qualitatively different distributions at week 3 (Fig. 5B) and week 6 (Fig. 5D), but display quantitative similarity in the averages: ∼70% have 1100 surface-VEGFR2/tumor cell at week 3 and ∼50% have 1000 surface-VEGFR2/tumor cell at week 6. The low-density subpopulations at these time points correspond to moderately low surface-VEGFR2: At week 3, ∼30% of the tumor cells have 3000 VEGFR2 on the tumor plasma membrane (Fig. 5B), while at week 6, 49% have only 1600 VEGFR2 on the cell surface (Fig. 5D). Very small populations are noted as having high receptor levels: at week 3, 7% of tumor cells have 17,900 surface-VEGFR2/tumor cell, and at week 6, 2% of tumor cells have 19,700 surface-VEGFR2/tumor cell.

### Characterizing autofluoresence

Our next objective was to estimate how autofluorescence affects our cell-by-cell data. For the tumor cells, autofluoresence data are examined by a cell-by-cell compilation of PE channel autofluorescence from nonlabeled cells and converted to numbers of VEGFRs/cell, using the PE calibration beads. For the tECs, the autofluoresence data are represented by a cell-by-cell compilation of PE channel fluorescence from tECs labeled with FITC and converted to numbers of VEGFRs/cell, using the PE calibration beads. A lognormal distribution fit well to each of the four autofluoresence datasets (Fig. 6A–D). The goodness of fit is further displayed by the representative lognormal probability plot for autofluoresence small tEC at week 3 (Fig. 6E). As such, FITC labeling has a spectral bleed-through into the PE channel representing an average of 464 VEGFRs/tEC at week 3 (Fig. 6A and Table 2) and 388 VEGFRs/tEC at week 6 (Fig. 6B and Table 2). Nonlabeled cells have autofluorescence levels corresponding to 590 VEGFRs/tumor cell at week 3 (Fig. 6C and Table 2) and 354 VEGFRs/tumor cell at week 6 (Fig. 6D and Table 2). The corresponding parameters, means and standard deviations, are given in Table 2. These data provide insight into the autofluorescence contribution to the cell-by-cell mixture means. For example, the tEC at week 6 are represented by a three-component lognormal mixture model of which, one population, comprising 52% of the cells has a mean of 600 VEGFR1/tEC, a second population, comprising 36% of the cells has a mean on 1300 VEGFR1/tEC, and a third population, comprising 12% of the cells has a mean of 10,600 VEGFR1/tEC. Based on autofluorescence values of 388 VEGFRs/tEC, 52% of the cells may have a mean of ∼200–600 VEGFR1/tEC, 36% of the cells may have a mean of ∼900–1300 VEGFR1/tEC, and 12% of the cells may have a mean of ∼10,200–10,600 VEGFR1/tEC.

**Table 2:** Parameters for autofluorescence data distributions.

	Mean (*μ*)	Standard deviation (*σ*)
Tumor endothelial cells (tEC)		
Week 3	464	2
Week 6	388	2
Tumor cells		
Week 3	590	1
Week 6	354	2

### Ensemble-averaged VEGFR-surface levels

**Figure 7:**
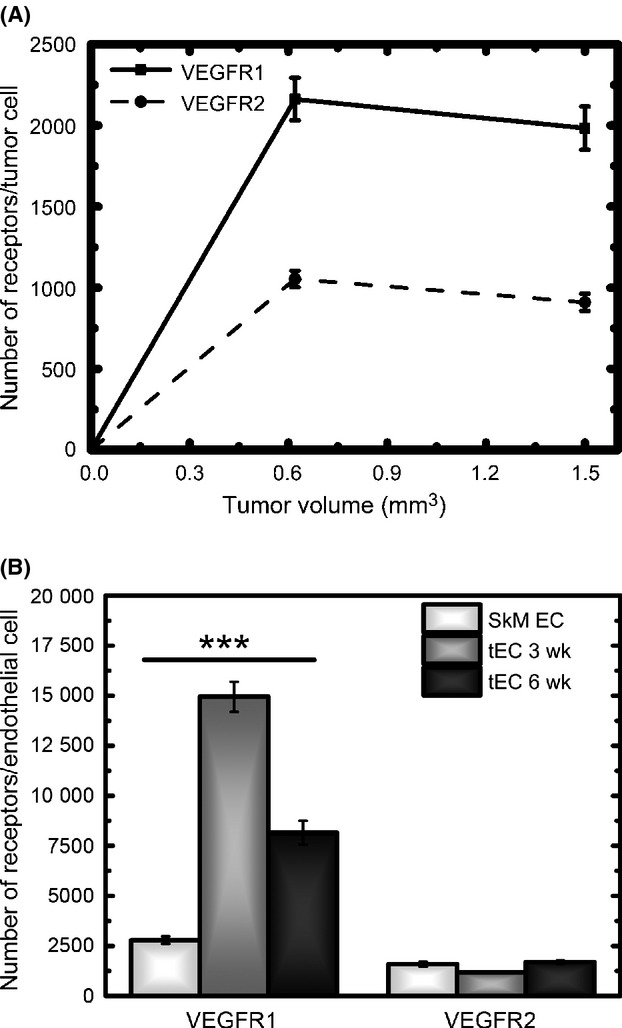
Ensemble-averaged time course of vascular endothelial growth factor receptor (VEGFR) surface distribution. (A) Tumor cells show significantly higher levels of VEGFR1 compared to VEGFR2. For a given VEGFR, the surface levels are not significantly different early in tumor development, 3 weeks, compared late in tumor development, 6 weeks. (B) Tumor endothelial cells show significant differences in VEGFR1 surface levels early in tumor development, 3 weeks, with nearly ∼15,000 VEGFR1/tEC. These levels are nearly halved late in tumor development, 6 weeks. The levels of VEGFR1/tEC at 3 and 6 weeks are significantly different from one another, and are also significantly different from the levels of VEGFR1 found to endothelial cells obtained from normal mouse skeletal muscle. The levels of VEGFR2 on tumor endothelial cells are not significantly different at 3 and 6 weeks, or on endothelial cells isolated from normal mouse skeletal muscle.*P < 0.05; **0.001< *P* <0.01; ***P < 0.001.

Although it is apparent that the isolated cells display significant heterogeneity, trends can be observed from the ensemble-averaged data. The large cells show: consistently low levels of VEGFRs; tECs having a higher average surface-VEGFRs compared to the tumor cells; and the highest VEGFR-surface levels are seen at week 6 (Table 3). The small cells have a higher average level of VEGFRs compared to the large cells (Tables 3 and 4). The small tEC have a higher average surface-VEGFRs compared to the small tumor cells (Table 4). VEGFRs on the small tumor cells remain approximately constant at weeks 3 and 6 (Fig. 7A). However, early-stage tumors (week 3) present significantly higher surface VEGFR1 on small tEC, ∼15,000 surface-VEGFR1/tEC when compared to week 6, where VEGFR1 surface levels are reduced by 45% on small tEC to 8150 surface-VEGFR1/tEC (Fig. 7B). Overall, the average levels of VEGFR2 on small tEC are between ∼1200 and 1700 surface-VEGFR2/tEC at weeks 3 and 6 (Fig. 7B), and they are in fact similar to the surface levels of VEGFR2 on endothelial cells derived from mouse skeletal muscle, ∼1100–1700 surface-VEGFR2/EC 41.

**Table 3:** Ensemble-averaged receptor statistics (large cells).

Sample	*n*	Mean ± SEM
VEGFR1		
Large human tumor cells		
Week 3	10	160 ± 30
Week 6	10	370 ± 30
Large mouse tEC		
Week 3	10	490 ± 40
Week 6	10	850 ± 110
VEGFR2		
Large human tumor cells		
Week 3	10	130 ± 10
Week 6	10	230 ± 20
Large mouse tEC		
Week 3	10	260 ± 30
Week 6	10	610 ± 80

VEGFR, vascular endothelial growth factor receptor; tEC, tumor endothelial cells.

**Table 4:** Ensemble-averaged receptor statistics (small cells).

Sample	*n*	Mean ± SEM
VEGFR1		
Small human tumor cells		
Week 3	10	2160 ± 130
Week 6	10	1980 ± 130
Small mouse tEC		
Week 3	10	14,950 ± 750
Week 6	10	8150 ± 600
VEGFR2		
Small human tumor cells		
Week 3	10	1050 ± 60
Week 6	10	910 ± 70
Small mouse tEC		
Week 3	10	1170 ± 40
Week 6	10	1690 ± 80

VEGFR, vascular endothelial growth factor receptor; tEC, tumor endothelial cells.

## Discussion

VEGFRs are the initial elements in rendering the extracellular VEGF signal to an intracellular response. As such, their surface levels significantly control angiogenic signaling. The isolation of mouse endothelial cells and human tumor cells from tumor xenografts, and the profiling of these cells thus represent an important step toward understanding the angiogenic receptor-mediated signaling balance. Our results show little to no VEGFR-surface expression on MDA-MB-231 cells, in vitro. We show that a HUVEC-saturating dose of 1 nmol/L VEGF-A_165_ cannot regulate VEGFR-surface levels on MDA-MB-231, in vitro. However, when the MDA-MB-231 cells are implanted into nude mice, the resulting xenografts have significant surface expression of both VEGFRs on tumor cells and tEC, both early and late in tumor development. We observe that the tumor cells and tEC are represented by multiple populations of cells, characterized by size and surface expression of a corresponding VEGFR. We find that all cells have higher surface expression of VEGFR1 compared to VEGFR2. We also find that the tEC have higher surface levels of VEGFRs compared to tumor cells.

### Statistical modeling of cellular distributions

It is well-established that cells exhibit autofluorescence due to intrinsically fluorescent molecules, primarily aromatic amino acid-containing molecules in mitochondria and other organelles 56. One such set of molecules include oxidized flavins, which have a broad-emission spectrum that overlaps with PE, with maximal flavin emission occurring at 520 nm 56. Given this overlap, it is especially important to characterize autofluorescence and interpret its contribution to the quantitative measurements. Furthermore, when using two or more fluorescent probes, the emission spectral overlap will affect signal intensity. In our studies, we used FITC as a secondary marker of tECs, and we used PE as our quantitative fluorescent probe on both tumor cells and tECs. The emission spectra of FITC and PE do overlap. As a result, we combined our analysis of tEC autofluorescence and FITC spectral bleed-through by quantifying the fluorescence intensity in the PE channel of tECs labeled with FITC: thus representing tEC autoflourescence data. When autofluorescence in the PE emission channel is converted to numbers of receptors/cell, the mean range can be ∼400–600 VEGFRs/cell, with autofluorescence depending on the cell type and the weeks of tumor growth, with higher autofluorescence being seen at week 3 across both cell types tested. Currently, there are no accepted metrics for analyzing autofluorescence data in the context of cell-by-cell quantitative fluorescence studies. Therefore, this analysis of autofluorescence provides an important paradigm for accounting for autofluorescence in quantitative fluorescence studies.

### MDA-MB-231 characteristics

In these studies, we examined the in vitro and tumor characteristics of MDA-MB-231 cells. These are a highly metastatic, breast cancer cell line obtained from a pleural effusion 57. They are defined as “Basal B,” due to their dendritic-like or stellate structural appearance, stem cell-like characteristics, and preferential expression of such genes as: CD44, MSN, and TGFBR2 58. These cells would clinically reflect a “triple-negative” tumor, lacking estrogen receptor (ER), progesterone receptor (PR), and epidermal growth factor receptor 2 (Her2/neu) receptors 58–61. Triple-negative tumors are more aggressive, being associated with poorer outcomes, following chemotherapy. These patients exhibit a higher relapse and a shorter life span 62–64. As such, understanding the biology of these types of tumors represents an urgent, translational need 65.

The aggressiveness of these types of tumors has led to the theory that these MDA-MB-231 cells have lost their responsiveness to normal cues in culture. In fact, one study found VEGFR2 to be constitutively phosphorylated on MDA-MB-231 cells, in vitro, and a 30 ng/mL VEGF dose does not change VEGFR2 phosphorylation 66. Our data on MDA-MB-231 cells, in vitro, showed no change in VEGFR surface levels with similar doses of VEGF (1 nmol/L or ∼25 ng/mL); whereas, we have previously shown that this dose over a 24 h period results in a 2.75-fold increase in VEGFR1 surface levels and a 4.6-fold decrease in VEGFR2 surface levels 22. Absence of MDA-MB-231 invasiveness has also been noted in a matrigel chemotaxis assay at 435 pmol/L–4 nmol/L VEGF 67. However, MDA-MB-231 cells are not completely unresponsive to VEGF: NRP1, which we 22 and others 68–70 have shown to be highly expressed on MDA-MB-231 cells, mediates VEGF-induced invasiveness 68. Furthermore, TGFβ-1 71, and serine proteases 72 can increase the production of VEGF; and IGF-1 can upregulate VEGF-C 73.

In addition to the in vitro responsiveness of MDA-MB-231 to growth factor, MDA-MB-231 in vitro expression of VEGFRs remains unresolved. Here, we showed little to no VEGFRs on the cell surface in vitro, robust VEGFR-surface expression on xenografts, and reduced VEGFRs when these cells are returned to two-dimensional culture. Some in vitro studies report little to no VEGFR protein and gene expression 69,74,75, others report moderate to robust VEGFR expression 66–68,75,76, and one group reports significant intracellular VEGFR1 localization 77. However, VEGFRs have been shown to be expressed on tumor through immunohistochemical staining of xenografts and on breast cancer tissue samples 74,78–83. Our results and those of others may be attributed to differing MDA-MB-231 phenotypes across laboratories, signifying the need for standardization of cell growth conditions. It is well-established that culture substrates, media components such as serum, and overall treatment of tumor cells can change their gene expression and functional profiles 84,85. Furthermore, as these receptors are certainly seen in three-dimensional tumors, it remains necessary to develop and adopt three-dimensional culture approaches, which represent the tumor microenvironment.

Indeed, when MDA-MB-231 cells are cocultured with endothelial cells, VEGF can induce MDA-MB-231 cell migration and adhesion, while the same study found that MDA-MB-231 cells alone are unresponsive to VEGF in an invasion assay 67. Another study showed that MDA-MB-231 cells grown as spheroids, do not exhibit the adhesive phenotype seen by the less invasive breast cancer cells MCF7 and MCF10A; however, adhesion of MDA-MB-231 spheroid-grown cells to E-Selectin can be induced by treatment with plasma, interleukin 6, or tumor necrosis factor alpha 86. Altogether, these studies indicate a need for continued development of 3D tumor cultures and continued mapping of the mechanism mediating tumor cell responses.

### Cell isolation

We have isolated both human tumor cells and mouse tEC, using a multistep approach that includes: enzymatic dissociation of tumor, binding of biotinylated CD31 antibody to cells, streptavidin-coated magnetic beads binding to antibody, and magnetic separation of this endothelial-enriched population. We then apply CD34-FITC labeling during flow cytometry to select the endothelial population, and exclude nonendothelial CD31^+^ cells. We have previously utilized this approach to isolate endothelial cells from skeletal muscle, and we have rigorously tested this approach to ensure preservation of VEGFRs during enzymatic tissue dissociation 41, enrichment of endothelial cells 24, antibody specificity, antibody saturation, and monomeric antibody binding 22. This is the first application of our optimized isolation approach to tumor specimens.

We observe that the isolates contain several subpopulations of tumor cells and tEC: one population of large cells with low levels of VEGFRs; three populations of small cells with low-VEGFR1, mid-VEGFR1, or high-VEGFR1 surface levels; and two populations of small cells with low-VEGFR2 or high-VEGFR2 surface levels. The presence of cells with low-VEGFR presentation correlates well with immunohistochemistry studies that have reported nonuniform VEGFR expression patterns on tumor vasculature. One study, which quantitatively compared VEGFR2 vascular staining to CD31 surface protein levels found that only 30% of tumor vessels have VEGFR2 79. Another study indicated that only 53% of tumor endothelium have VEGFR1 and only 47% of tumor endothelium have VEGFR2 82. Yet another found very low levels of VEGFR1, 16%, in ductal carcinoma in situ, and 53% tumor expression of VEGFR2 87. Our studies provide a more sensitive method of determining how these receptors are presented. Through cell-by-cell analysis, we showed that a mixture model best represents the small cell population, which comprises cells with low-surface expression, mid-surface expression, and high-surface expression. Furthermore, this mixture of endothelial cells further characterizes the heterogeneity in tumor vasculature. Vascularization in tumors shows marked departures from physiological vessel architecture: increased leakiness and tortuosity, decreased pericyte coverage, and abnormal organization 88,89. Indeed, the heterogeneity that we observe in the tEC, defined by the presence of multiple subpopulations surpasses what we have previously reported with regards to surface-VEGFRs on endothelial cells in vitro, ex vivo, and even on ischemic mouse endothelial cells ex vivo; wherein, we only observed one population of cells. Due to the nature of our profiling, one read-out per cell, we do not have knowledge of any population overlap across cells expressing differing levels of VEGFR1 and VEGFR2; however, this underlines a need for multicolor imaging approaches to better characterize these heterogeneities.

### VEGFR-surface expression

The highest surface-receptor expression pattern is observed early in tumor growth (3 weeks), with the tEC having an average of nearly 15,000 VEGFR1/tEC. This represents substantial surface expression, which is significantly decreased later in tumor development. Although the ensemble averaged data provide significant insight into the data trends, we observe that artifacts can be introduced through averaging. More specifically, the cell-by-cell profiling shows that at week 6, a majority of the tEC have low levels of VEGFR1, <1000 VEGFR1/tEC, 36% display a mean of 1300 VEGFR1/tEC, while only 12% of cells have an average of 10,600 VEGFR1/cell; however, the ensemble average represents ∼8000 VEGFR1/tEC. Thus, the cell-by-cell profiling reveals a high-density population of low-expressing cells, which are not fully represented by the mean.

The population of endothelial cells with high surface levels of VEGFR1, early in tumor development, raises the question of the functional role of VEGFR1 in tumors. VEGFR1 exhibits both proangiogenic and antiangiogenic properties. VEGFR1 may serve as a positive regulator under pathological conditions, where its expression may promote angiogenesis 90. VEGFR1 may also serve as a negative regulator both through downregulation of VEGFR2-mediated signaling 91 and due to its 10-fold higher affinity for VEGF, compared to VEGFR2, but low tyrosine kinase activity 92,93. One study has found that VEGFR1 expression on patient breast cancer tumor cells, following neoadjuvant chemotherapy, is correlated with increased survival; in fact, the number of tumors expressing VEGFR1 increased by nearly 25% following therapy 94. While a conflicting result found that VEGFR1 is associated with higher metastatic risk in breast cancer 95.

Despite the lack of consensus on the prognostic role of VEGFR1 in tumor, the high surface levels of VEGFR1 that we observe early in tumor development suggest that antiangiogenic therapies may need to be tuned to respond to the dynamics of the progressing tumor microenvironment. Our recent work on the mouse hindlimb ischemia model has also shown changing VEGFR distribution on endothelial cells at different stages of the mouse hindlimb ischemia model: 40% higher VEGFR1 on endothelial cells from ischemic hindlimb 10 days after femoral artery ligation 24. Together, these data support the theory that patient sensitivity to antiangiogenic therapy may correlate to tumor stage 96,97. Furthermore, these data support the pedagogy that a single agent, such as anti-VEGF therapy, responding to the high-VEGF found in patient serum 52 may not be as effective as a dynamic therapeutic approach that responds to the progressing tumor microenvironment. A dynamic therapeutic approach would have a goal of reducing angiogenic signaling through the upregulated VEGFR1. At present, it would be difficult to correlate the increased VEGFR1 surface levels to increased second messenger signaling, as VEGFR1 signals through the PI3K/Akt and phospholipase C gamma/protein kinase C pathways 98, which are constitutively active in breast cancer cell lines, xenografts, and tissue 66,99–102. To examine the utility of early tumor therapy targeting VEGFR1, future studies should combine it with profiling of tumor signaling tumor vasculature, and angiogenic receptors.

### Proteomic profiling and breast cancer

Cell types are classically defined by their surface expression of specific cluster of differentiation (CD) markers 103–106; and in cancer, tumor subtypes are defined by their expression of cell surface receptors, including HER+ and PR+ in breast cancer 107. Therefore, the quantity and distribution of receptors on the cell surface significantly affects intracellular signaling while serving as a defining characteristic of cells. However, surface proteomics has not typically not mapped—despite these being the primary conduits of extracellular to intracellular signal transduction. These limitations in surface proteomics are very much due to the lack of tools for studying membrane proteomics. Flow cytometry offers a useful approach to profile cell surface expression; however, on its own it provides only a relative indication of receptor levels. The use of quantitative flow cytometry represents a shift in the current research paradigm, providing absolute levels of cell surface proteomics. Here, we show that these proteomic profiling studies can identify the presence and quantity of receptors on isolated cells.

Cell surface proteomic profiling also offers a useful approach to characterizing tumor heterogeneity. Tumor heterogeneity represents a challenge in the emerging field of personalized medicine 108,109 and a grand challenge in interfacing engineering with the life sciences 110. Variability in patient populations can result in differential therapeutic outcomes 31. Similarly, genetic screens of patient tumor samples have highlighted the challenge of tumor heterogeneity in both personalized medicine and biomarker development, finding intratumor mutational disparities in renal cell carcinoma patient biopsies 111. These patient-to-patient challenges indicate a need to better characterize and understand tumor heterogeneity. As such, this profiling technology can be further extended toward defining heterogeneity in patient tumor samples.

Quantitative receptor profiling of patient samples would speed progress into personalized medicine methodologies, with significant advancement when the patient profiling is coupled to computational modeling platforms. Our recently developed systems biology framework, which utilizes a bimodal, experimental and computational methodology, predicted the optimal drug characteristics for which therapeutic angiogenesis agents may have an advantageous effect 21. Similarly, these data we report, here, provide critical parameters needed for continued advancement of tumor modeling 21,112–115. Given the wealth of cell-by-cell data that we report, new models can be created that incorporate: cell-by-cell heterogeneity and tumor dynamics at early and late stages. Together these data and model expansion can be used to examine systemic changes and therapeutic responses; thus, advancing angiogenic targeting for predictive medicine.

This is the first study to characterize ex vivo VEGFR receptor localization in tumor, and as such, it provides critical insight into the abundance of these key signaling receptors within the tumor microenvironment. Furthermore, it establishes this profiling technology for future translation to patient tissue.

## Supporting Information

Figure S1 Fitting VEGFR1 tEC week 3 data to 2- and 3- component lognormal mixture models.
