# Supplementary figures and images for: Quantitative fluorescent profiling of VEGFRs reveals tumor cell and endothelial cell heterogeneity in breast cancer xenografts
Source: Cancer Med. 2014 Jan 22;3(2):225–44. doi: 10.1002/cam4.188 (PMC3987073; doi:10.1002/cam4.188)

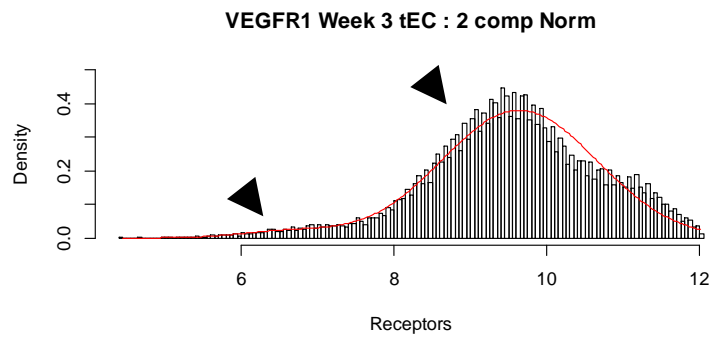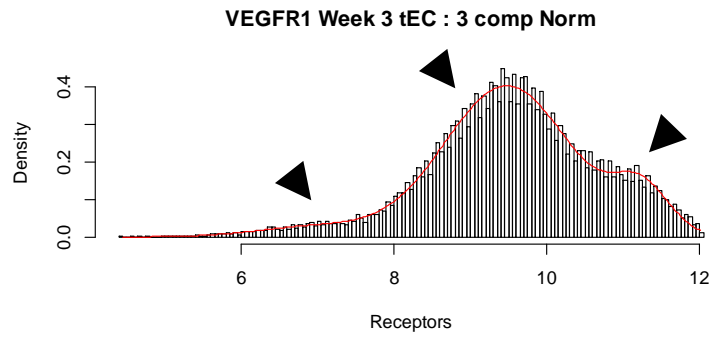

**Supplementary Figure 1**

Supplement: Figure S1 — Fitting VEGFR1 tEC week 3 data to 2- and 3- component lognormal mixture models. [file cam40003-0225-sd1.pdf]
